# Rotating Bending Fatigue Behaviors of C17200 Beryllium Copper Alloy at High Temperatures

**DOI:** 10.3390/ma16020815

**Published:** 2023-01-13

**Authors:** Fuqiang Lai, Kun Mao, Changsheng Cao, Anqiong Hu, Junxiang Tu, Youxi Lin

**Affiliations:** School of Mechanical Engineering and Automation, Fuzhou University, Fuzhou 350116, China

**Keywords:** beryllium copper alloy, elevated temperatures, rotating bending fatigue, fatigue strength, performance degradation

## Abstract

The purpose of this paper is to investigate the fatigue properties of C17200 alloy under the condition of quenching aging heat treatment at high temperatures, and to provide a design reference for its application in a certain temperature range. For this purpose, the tensile and rotary bending fatigue (RBF) tests were carried out at different temperatures (25 °C, 150 °C, 350 °C, and 450 °C). The tensile strength was obtained, and relationships between the applied bending stress levels and the number of fatigue fracture cycles were fitted to the stress-life (S-N) curves, and the related equations were determined. The fractured surfaces were observed and analyzed by a scanning electron microscopy (SEM). The results show that the RBF fatigue performance of C17200 alloy specimens is decreased with the increase in test temperature. When the temperature is below 350 °C, the performance degradation amplitudes of mechanical properties and RBF fatigue resistance are at a low level. However, compared to the RBF fatigue strength of 1 × 10^7^ cycles at 25 °C, it is decreased by 38.4% when the temperature reaches 450 °C. It is found that the fatigue failure type of C17200 alloy belongs to surface defect initiation. Below 350 °C, the surface roughness of the fatigue fracture is higher, which is similar to the brittle fracture, so the boundary of the fracture regions is not obvious. At 450 °C, due to the further increase in temperature, oxidation occurs on the fracture surface, and the boundary of typical fatigue zone is obvious.

## 1. Introduction

**Figure 1:**
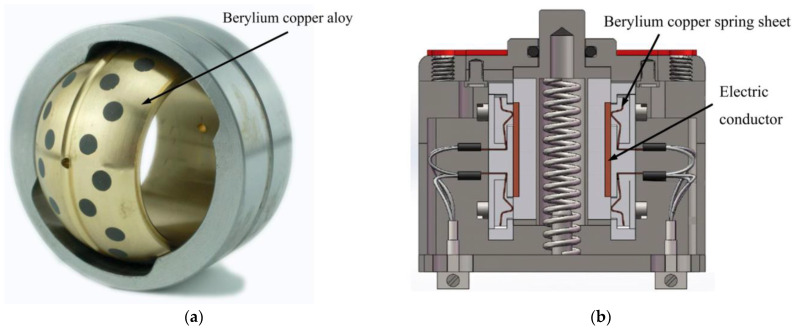
Typical mechanical components made of beryllium copper alloy. (**a**) Joint ball bearing; (**b**) high temperature slide switch.

Beryllium copper alloy is a type of precipitation hardening alloy that combines the properties of copper and beryllium with high tensile strength limit, elasticity, and fatigue resistance, as well as high electrical conductivity, wear, and corrosion resistance [1]. For instance, beryllium copper alloy was utilized in joint ball bearing because of its high wear resistance (Figure 1a). Furthermore, beryllium copper alloy is used in many instrumentations due to its superb mechanical strength and elasticity, including the aerospace precision instruments, as well as high-temperature electric switches and electric connectors, as shown in Figure 1b.

With the further development of the application of beryllium copper alloy, the research on the factors affecting its mechanical strength and fatigue mechanism is becoming more and more thorough. Many scholars conducted the fatigue property evaluation and fatigue mechanism analysis of beryllium copper alloys.

The content of elements has great influence on the mechanical strength and fatigue properties of beryllium copper alloys. For example, Pan et al. [2] investigated the effects of Ni, Co, Mg, Ti, and other added elements and impurity elements, such as Fe and Si, on the microstructures and mechanical properties of beryllium bronze sheet material, and they found that the fatigue resistance of beryllium copper alloy, with the addition of trace amounts of magnesium, was improved, and there was a significant increase in tensile strength, yield strength, elastic limit, and hardness.

Fracture and fatigue of material is an irreversible process and the failure process and mechanism were the focus of many researchers in various application conditions of beryllium copper alloys. For instance, in the aspect of the fatigue performance assessment of beryllium copper alloy, Fan et al. [3] conducted a comparative study on the bending fatigue performance of beryllium copper sheets from three manufacturers, and the fatigue resistance of the three products was compared and analyzed by using the group comparison tests method. In addition, Pang et al. [4] systematically investigated the high-cycle fatigue behaviors of beryllium bronze in the tensile strength range of 500 MPa to 1300 MPa, and the relationships between fatigue properties and the microstructure of beryllium bronze were discussed, especially the relationships between the size of precipitate and grains. The results show that the contribution of fine-grain strengthening to fatigue performance improvement is higher than solid solution strengthening and precipitation strengthening. Wang et al. [5] performed fatigue fracture failure analysis on beryllium copper spring components. They reported that beryllium copper springs fractured at low operating frequencies due to coarse second-phase precipitate grains, resulting in a small grain size and a low hardness. Zhu et al. [6] conducted rotating bending fatigue (RBF) experiments on hardened ageing beryllium copper alloy rods under different stress amplitudes at room temperature, they found that rotating bending fatigue cracks alloy rods under different stresses (440 MPa to 600 MPa) initiated at the surface defects of the specimens, and the cracks consisted of three regions: the crack sprouting region, the crack extension region, and the final fracture region. Subsequently, the fatigue fracture mechanisms of beryllium copper alloy were then discussed. They found that the second phase and inclusion particles near the surface of the sample would lead to the initiation of cracks in the weak spot.

Service temperature is a sensitive factor of influencing metal and alloy fatigue performance, and the fatigue properties of copper alloys will be decreased significantly at elevated temperatures. Many scholars conducted studies in this research area. For instance, Li et al. [7] summarized some expressions that can provide quantitative evaluation of the thermal creep and fatigue life of various copper alloys. Miura et al. [8] investigated the temperature dependence on the cyclic creep behavior of Cu-SiO_2_ bi-crystals of different controlled misorientation angles. Berto et al. [9] studied the high-temperature fatigue strength of copper–cobalt–beryllium alloys.

Based on the excellent properties of beryllium copper alloy, it can be used to manufacture small and complex shape mechanical parts with high resistance to contact stress. In addition, the excellent electrical conductivity can counteract the high impedance caused by miniaturized circuits, and the good stress relaxation performance ensures a long-term trouble-free operation, even at high-temperature work conditions. Hence, with the further development of beryllium copper alloy, it was introduced to many special industry application fields.

For example, in the space field engineering application, a spacecraft or space station needs to withstand a high amplitude of temperature variation in space, and the side facing the sun needs to endure heat (sunlight) for dozens of hours. High requirements of service reliability for instruments and components used in space filed in high-temperature environments are proposed; however, there is little published research in the area of beryllium copper alloy service performance at elevated temperatures, and there is a lack of reference for reliability design in the engineering applications of beryllium copper alloy. This paper aims to explore the fatigue life changes and fatigue mechanism analysis of C17200 alloy at different temperatures, and the corresponding experimental results and theoretical support for the application of C17200 alloy at different temperatures can be then provided.

**Figure 2:**
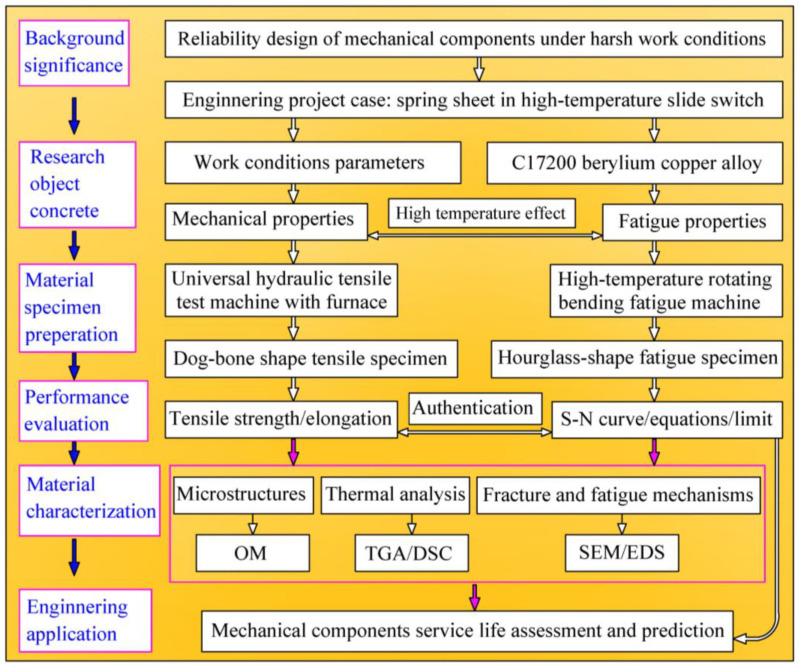
Research idea flow and contents arrangement.

Research idea flow and content arrangement of this study are illustrated in Figure 2. Based on the reliability design of mechanical components under the severe work conditions, a high temperature switch with a spring sheet is chosen as the engineering project case, and the spring sheet is manufactured of C17200 beryllium copper alloy. The high temperature effects on the mechanical properties and rotating bending fatigue properties of C17200 alloy will be evaluated and quantified. The authors aim to provide a reasonable reference with experimental results for the design of the mechanical component of beryllium copper alloy.

## 2. Materials Preparation and Experimental Details

### 2.1. Experimental Materials and Material Characterization

The C17200 beryllium copper alloys used in this study are squeezing bar materials with a diameter of 8 mm, and the chemical compositions are shown in Table 1. The bar materials were obtained from copper ingots through a series of preparation processes, including heating, forging, and drawing, and the heat treatment processes are solid solution, cold deformation, and aging heat treatment. The main mechanical physical property parameters of C17200 alloy are listed in Table 2.

The material specimens were prepared in accordance with the requirements of Chinese Standards ‘QJ 2337-1992 Metallographic test methods of beryllium bronze’ and ‘HB7694-2001 Metallographic analysis method of beryllium bronze’. Specimens of microstructure observation were firstly polished by grit sandpapers by hand, and the surface was then polished by a professional metallographic polishing machine until there were no obvious micro-scratches. Finally, the specimens were etched with an ammonia solution to copper dichloride as the etching agent. After the above processes was completed, an inverted metallographic optical microscope (MDS400, Wuhan Coulter Technology Co., Ltd., Wuhan, China) was used to observe the microstructures and the metallurgical photos were then obtained.

### 2.2. Mechanical Properties and Rotating Bending Fatigue Test Method

**Figure 3:**
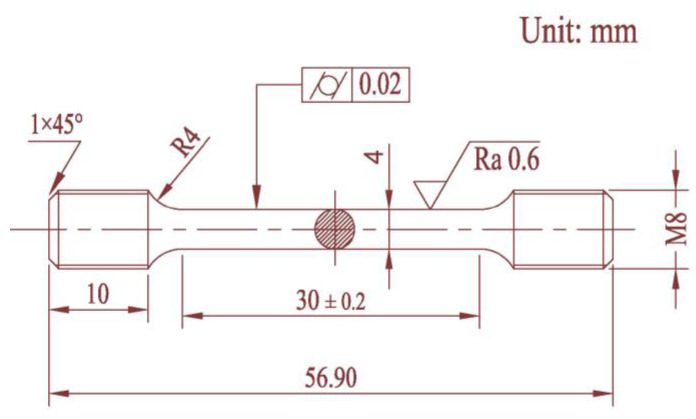
Specimen for tensile test and its dimensions.

**Figure 4:**
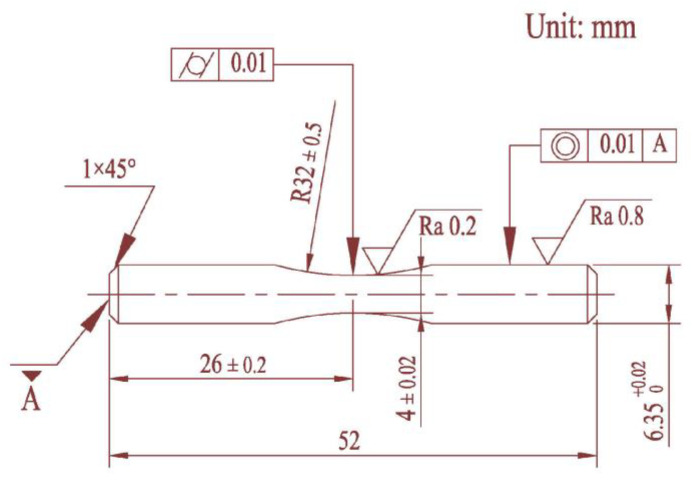
Specimen for fatigue test and its dimensions.

The experimental material was machined into symmetrical dog-bone-shaped tensile specimens and symmetrical hourglass-shaped rotating bending fatigue specimens. The tensile strength tests were performed by a universal hydraulic tensile test machine (WDW-200, Tianchen Test Co., Ltd., Jinan, China), and the specimens were tested and heated in a furnace. The specific size of the tensile sample is shown in Figure 3. Some materials were mechanical machined into hourglass shapes according to the Chinese Standard ‘GB/T 4337-2015 Rotating bending fatigue test method of metallic materials’, and the specific dimensions of fatigue specimen are shown in Figure 4. In order to reduce the influence of machining error on the fatigue test results, the concave arc section of all fatigue specimens was treated by grinding and polishing; dimensional accuracy could be then obtained.

**Figure 5:**
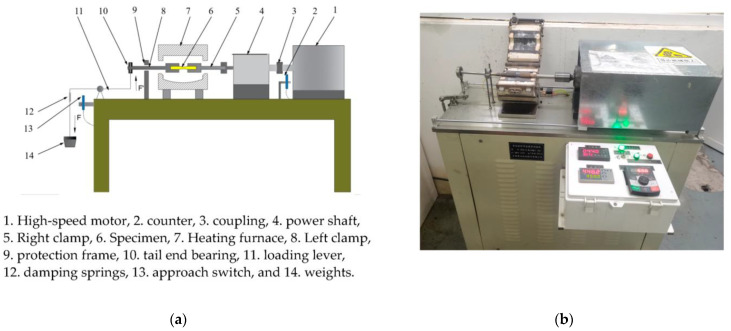
High-temperature rotating bending fatigue machine. (**a**) Schematic diagram and structural compositions of high-temperature rotating bending fatigue machine; (**b**) high-temperature rotary bending fatigue testing machine physical picture.

The fatigue tests were performed through utilizing a high-temperature rotating bending fatigue machine (GWXW-25, Jinan Jiacheng Precision Test Instrument Co., Ltd., Jinan, China), and the schematic diagram and structural compositions of the machine are illustrated in Figure 5. During the RBF tests, a stress ratio of R = −1 was used, and the loading spectrum was a sine wave with a frequency of 50 Hz. The bending stress level was supported by a loading system and determined by weights. The specimens were tested for rotating bending fatigue performance at 25 °C, 150 °C, 350 °C, and 450 °C, respectively.

After the RBF fatigue experiments, the fatigue fracture surfaces were observed and analyzed through utilizing a tungsten filament type scanning electron microscope and energy-dispersive spectroscopy (Quanta 250, FEI, Portland, OR, USA).

## 3. Experimental Results and Discussion

### 3.1. Microstructures

**Figure 6:**
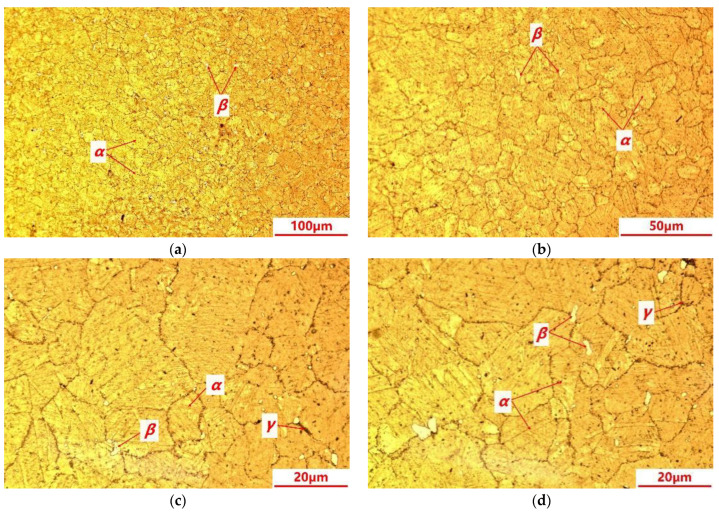
Microstructures of C17200 beryllium copper alloy. (**a**) Microstructure with magnification of 200×; (**b**) microstructure with magnification of 500×; (**c**) microstructure with magnification of 1000×; and (**d**) microstructure with magnification of 1000×.

The metallographic microstructures of the C17200 beryllium copper alloy are shown in Figure 6. The microstructures mainly consist of the supersaturated solid solution α phase, which is a solid solution of Be in Cu with a relatively uniform grain size, and the color of the α phase is darker and thus clearly distinguishable. The β phase is a disordered body-centered cubic solid solution with a good high-temperature stability, showing a bright white color under the erosion of the ammonia solution of copper chloride. It is observed that a large number of γ-reinforced phases are precipitated at the grain boundaries and inside the grains, and most of them are precipitated at the grain boundaries. The γ-phase is a solid solution based on the electronic compound CuBe, with an ordered body-centered cubic dot matrix, which is characterized as hard and brittle [11,12].

### 3.2. Thermogravimetric Analysis and Differential Scanning Calorimetry

**Figure 7:**
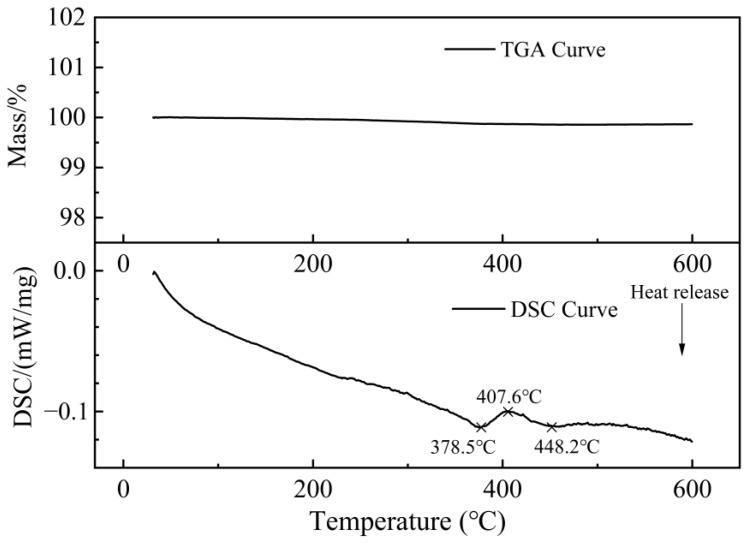
TGA/DSC curves of C17200 beryllium copper alloy.

A suitably sized sample is removed from the as receipt C17200 beryllium copper alloy bar and this sample is analyzed by a simultaneous thermal analyzer (STA449C/6/G, NETZSCH, Selb, Germany) with argon gas protection. Figure 7 presents the TGA/DSC measurement results of C17200 alloy. The figure is divided into two parts, and the horizontal coordinate is the set temperature (°C). The upper curve t is results of thermogravimetric analysis (TGA), and the lower curve is the results of differential scanning calorimetry (DSC). As a whole, the TGA curve tends to be straight with a much lower slope value, indicating that the sample weight was nearly unchanged during the whole test, and no obvious oxidation reaction occurred on the sample. The DSC curves show that the sample was exothermic until about 380 °C, accompanied by the release of residual stresses generated during the preparation of sample. After 378.5 °C, it is found that the heat absorption starts, with a peak at 407.4 °C, and the whole absorption peak ends at about 450 °C, during which the phase transition of C17200 alloy occurs, resulting in the variation in material performance.

### 3.3. Tensile Properties at Different Temperatures

**Figure 8:**
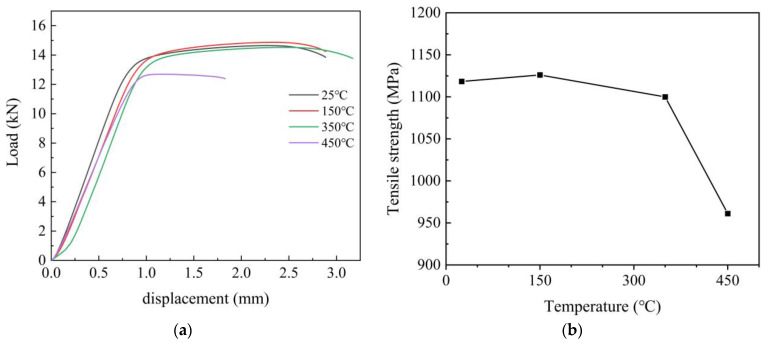
Load displacement curves and tensile strength of C17200 beryllium copper alloy at different temperatures. (**a**) Load displacement curves at different temperatures; (**b**) tensile strength at different temperatures.

Figure 8 shows the load-displacement curves and the tensile strength results of the C17200 alloy at different test temperatures, respectively. It can be seen from Figure 8a that the tensile length of C17200 alloy is very close to that of the alloy between 25 °C and 350 °C, and the tensile load limit and tensile value of the alloy decrease significantly at 450 °C. In Figure 8b, it can be seen that the variation amplitude of ultimate tensile strength is at a low level when tested at 25 °C, 150 °C, and 350 °C; however, the tensile strength is significantly decreased when the test temperature reaches 450 °C. It can be inferred that the decrease in tensile strength should be attributed to the phase transition and material performance degradation result from the increase in external temperature (heating). The tensile test process of the C17200 alloy could be regarded as a typical rheological process [13]. It is also consistent with the results of the TGA/DSC results discussed in Section 3.2. Based on the above analysis, it can be found that the mechanical properties of C17200 alloy are decreased with the increase in test temperature in the range of 25 °C to 450 °C, which would influence the service performance, including fatigue resistance. Moreover, the enhancement of toughness could improve the fatigue properties of copper alloys to a certain extent [14].

**Figure 9:**
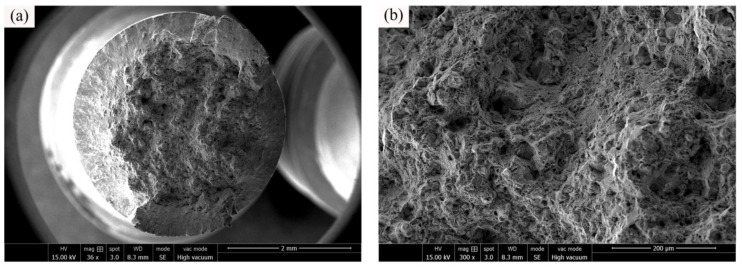

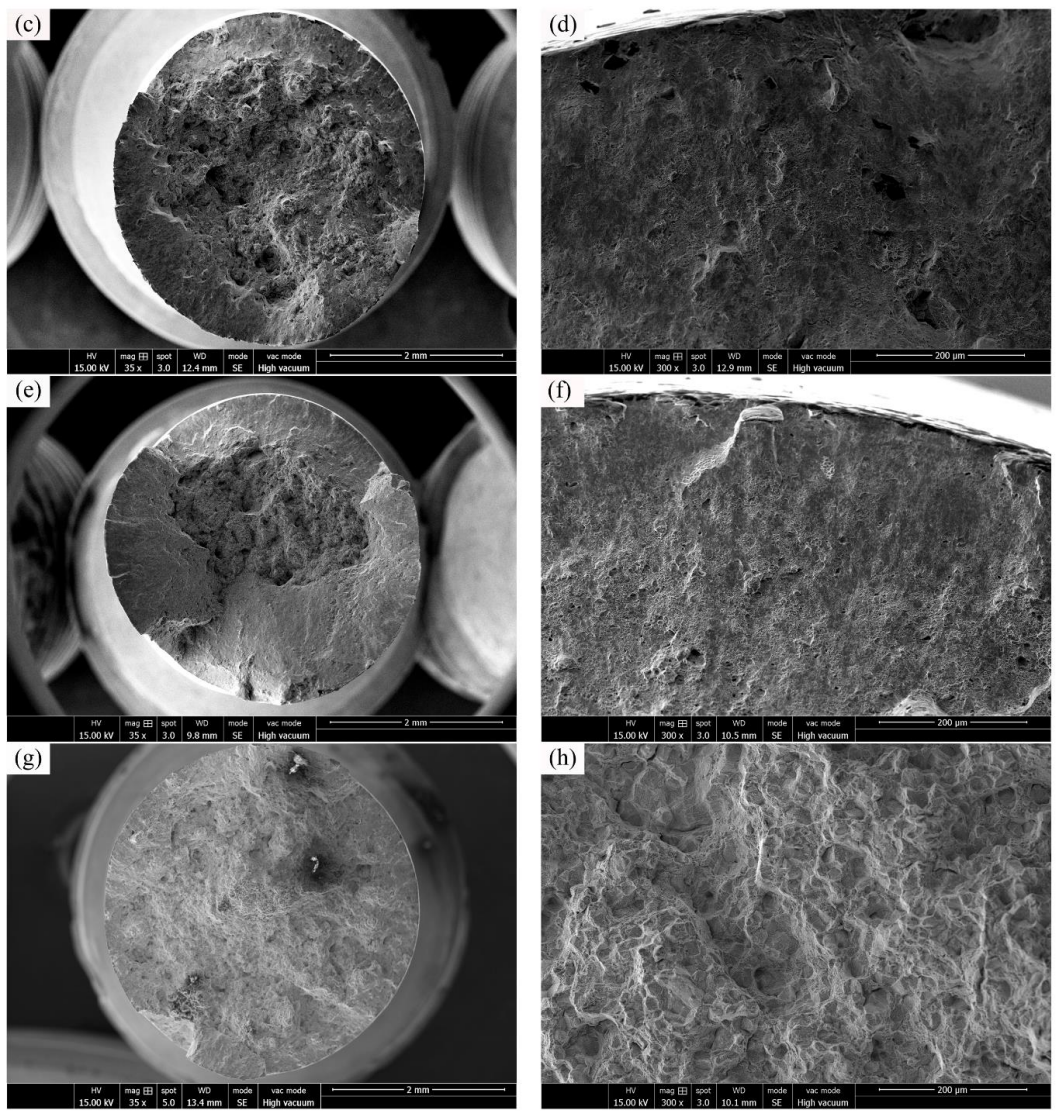
Tensile fractured surfaces of C17200 beryllium copper alloy tested at different temperatures. (**a**) General view of tensile fracture surface at 25 °C; (**b**) central fracture area morphology at 25 °C; (**c**) general view of tensile fracture surface at 150 °C; (**d**) topography of sliding zone at 150 °C; (**e**) general view of tensile fracture surface at 350 °C; (**f**) topography of sliding zone at 350 °C; (**g**) general view of tensile fracture surface at 450 °C; and (**h**) central fracture area morphology at 450 °C.

In order to investigate the tensile fracture mechanisms, the surface morphologies of C17200 alloy tensile test specimens were analyzed and represented in Figure 9. Generally, the fracture mechanism of the specimens belongs to a mixed tough-brittle fracture mode, and the fracture morphologies [15] at different temperatures show different material failure characteristics. It can be observed from the overall figure of the fracture in Figure 9 that the fracture edge of the sample has obvious slip line characteristics, indicating that obvious plastic deformation occurred before the fracture of the sample. This is caused by the grain sliding along the grain boundary under the action of shear force with the increase in sample deformation. There are many cleavage steps and cleavage planes in the center area of the fracture, and the river pattern is generated by the convergence of cleavage planes, so the center area of the fracture behaves a brittle fracture. Comparing to the morphologies of the fracture center area in Figure 9b,h, it can be seen that the fracture tends to be flat after the increase in tensile test temperature, and the number of dimples is decreased. Comparing Figure 9a–e, it can be seen that the fracture slip area is increased at 350 °C, indicating that the plasticity of C17200 alloy would be increased with a certain extent at 350 °C. In general, alloys with a high yield strength could obtain a better fatigue performance. In fact, the fatigue properties of alloys mainly depend on the elastic part of the strain range [16].

### 3.4. S-N Curves at Different Temperatures

In the design of a structure component or machine, fatigue reliability analysis is based on economic principles to evaluate the normal working conditions of the product of a certain period of use. It is required to provide reliable fatigue performance assessment results. Given the high dispersion level of fatigue test results, it is necessary to use statistical methods to evaluate and determine the S-N curve, so as to estimate the fatigue strength corresponding to a given fatigue life or cycles.

Stress-life curve, also known as S-N curve, taking the fatigue strength of material as the vertical coordinate and the logarithm value of fatigue life, is determined as the horizontal coordinate. S-N curves represent the relationships between fatigue strength and fatigue life of materials.

Based on the experimental results obtained from the RBF tests of C17200 beryllium copper alloy specimens at 25 °C, 150 °C, 350 °C, and 450 °C, respectively, the S-N curve would be is fitted statistically by using a three-parameter power function [17,18,19]. The formula of the three-parameter power function is expressed as Equation (1).

(1) $${\left(S_{max}-S_{0}\right)}^{m}N=C$$

where *S_max_* indicates the maximum stress, *N* indicates the fatigue life, and *m* and *C* represent the material properties parameters.

The undetermined constant in the equation can be obtained by the following method.

As reported by Gao et al. [20], taking the logarithm of each side of Equation (1), Equation (2) could be then obtained.

(2) $$lgN=lgC-mlg\left(S_{max}-S_{0}\right)$$

Letting *A*_1_ = *lgC*, *A*_2_ = −*m*, and *A*_3_ = *S*_0_, Equation (2) is then simplified as expressed in Equation (3).

(3) $$lgN=A_{1}+A_{2}lg\left(S_{max}-S_{0}\right)$$

Letting *X* = *lgN*, $Y=lg\left(S_{max}-S_{0}\right)$, Equation (3) is then simplified as expressed in Equation (4).

(4) $$X=A_{1}+A_{2}$$

There is a linear relationship between variables *X* and *Y* in Formula (4), so a set of corresponding data (*Xi*, *Y_i_*) (*i* = 1 ,2 ,⋯, n) can be obtained according to a known set of test data (Ni, S_i_) (*i* = 1 ,2 ,⋯, n). Then, the undetermined coefficients *A*_1_, *A*_2_, and *r* are determined by linear regression analysis.

(5) $$A_{1}=\bar{X}-A_{2}\bar{Y}$$

(6) $$A_{2}=\frac{L_{XY}}{L_{YY}}$$

(7) $$r=\frac{L_{XY}}{\sqrt{L_{XX}\;.\;L_{YY}}}$$

$\bar{Y}$, *L_YY_*, and *L_XY_* are all related to *S*_0_ and they are the functions of *S*_0._ Hence, *A*_1_, *A*_2_, and *r* are also the functions of *S*_0,_ which are *A*_1_(*S*_0_), *A*_2_(*S*_0_), and *r*(*S*_0_). The *S*_0_ should be obtained by maximizing the absolute value of correlation coefficient $\left|r\left(S_{0}\right)\right|$, hence, *S*_0_ could be obtained by the following equations:

(8) $$\frac{d\left|r\left(S_{0}\right)\right|}{dS_{0}}=0$$

(9) $$\frac{1}{L_{XY}}\cdot \frac{dL_{XY}}{dS_{0}}-\frac{1}{2L_{YY}}\cdot \frac{dL_{YY}}{dS_{0}}=0$$

According to Equations (8) and (9), *S*_0_ can be iteratively obtained by a method of dichotomy.

With the determination of *S*_0_, the values of *A*_1_, *A*_2_, and *r* can be obtained by Equations (5)–(7). Then, the values of *C* and *m* can be obtained by formulas *A*_1_ = *lgC* and *A*_2_ = −*m*.

Finally, based on the RBF test results, the fitted equation for the S-N curve of C17200 alloy at 25 °C is determined as shown in Equation (10):

(10) $${\left(S_{max}-415\right)}^{1.31516}N=7.54614\times 10^{8}$$

The fitted equation for the S-N curve of C17200 alloy at 150 °C is:

(11) $${\left(S_{max}-369.2\right)}^{2.4542}N=1.3490\times 10^{11}$$

The fitted equation for the S-N curve of C17200 alloy at 350 °C is:

(12) $${\left(S_{max}-340\right)}^{2.5739}N=3.8164\times 10^{11}$$

The fitted equation for the S-N curve of C17200 alloy at 450 °C is:

(13) $${\left(S_{max}-230\right)}^{2.9241}N=5.6310\times 10^{11}$$

**Figure 10:**
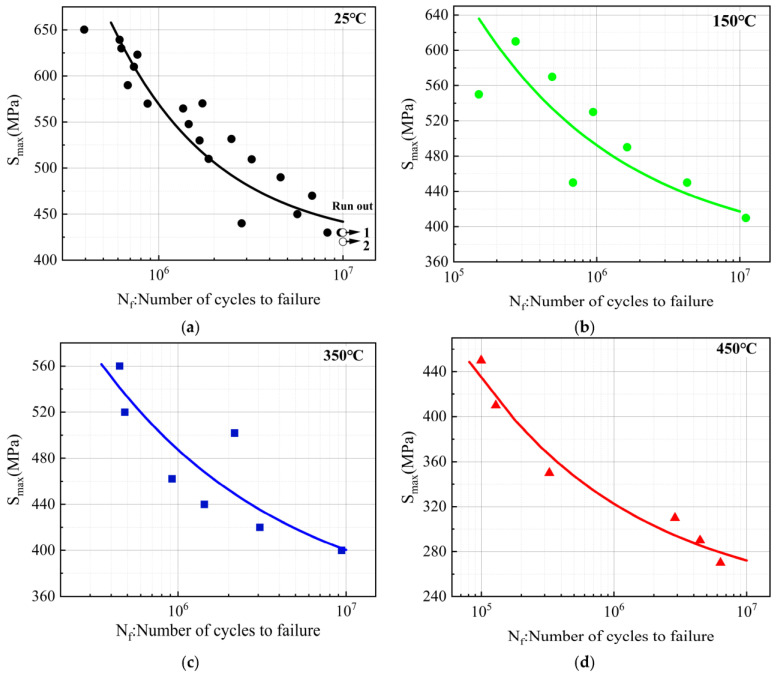

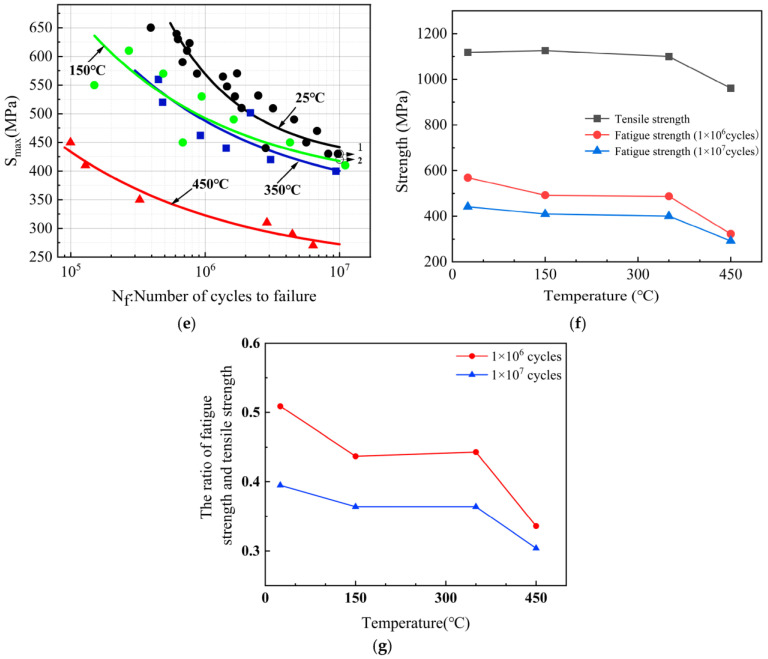
S-N curves of C17200 beryllium copper alloy at different temperatures. (**a**) S-N curve of C17200 alloy at 25 °C; (**b**) S-N curve of C17200 alloy at 150 °C; (**c**) S-N curve of C17200 alloy at 350 °C; (**d**) S-N curve of C17200 alloy at 450 °C; (**e**) S-N curves of C17200 alloy at different temperatures; (**f**) comparison of fatigue strength of C17200 alloy at different temperatures at 1 × 10^6^ cycles and 1 × 10^7^ cycles; and (**g**) ratio of fatigue strength to tensile strength of C17200 alloy at different temperatures.

The fatigue test data and the fitted S-N curves of C17200 alloy at different temperatures are shown in Figure 10. It can be seen that the fatigue life is gradually increased as the cyclic stress is decreased. The fatigue strength of the C17200 alloy specimens at 1 × 10^7^ cycles at 25 °C is 441.8 MPa. Based on the equations of the fitted curves, it can be determined that the fatigue strength (1 × 10^7^ cycles) of the beryllium copper alloy at other three test temperatures is about 409.4 MPa, 400.3 MPa, and 272.1 MPa, respectively.

It can be seen from Figure 10e that the fatigue resistance of C17200 alloy is nearly close at 150 °C and 350 °C. Compared to fatigue resistance at 25 °C, the fatigue strength (1 × 10^7^ cycles) at 150 °C and 350 °C is decreased by 7.3% and 9.4%, respectively. However, the fatigue strength (1 × 10^7^ cycles) of beryllium copper samples at 450 °C decreased by 38.4%. This could be due to the significant softening of beryllium copper alloy at 450 °C, leading to the severe degradation of material properties. C17200 alloy has a continuous phase transition between 400 °C and 450 °C, and the high-temperature environment results in a reduction in the force between grain boundaries. As shown in Figure 10f, it can be found that the ultimate tensile strength of C17200 alloy samples at different temperatures behaves the same variation trend as the fatigue strength at 1 × 10^6^ cycles and 1 × 10^7^ cycles, which would be decreased with the increase in fatigue test temperature. The decrease amplitude is at a low level below 350 °C, and the tensile strength and fatigue strength of beryllium copper samples are decreased significantly when the temperature reaches 450 °C. Figure 10g shows the ratio of fatigue strength and tensile strength of C17200 alloy at different temperatures. The general trend is that it is decreased with the increase in temperature, which can provide reasonable reference for reliability design of components in practical engineering applications.

### 3.5. Fatigue and Fracture Mechanisms Analysis

Metallic material fatigue failure generally has five models, including cracking caused by surface defects, cracking caused by near surface carbides, nonmetallic inclusions as sources, and cracking caused by carbides, as well as nonmetallic inclusions within materials [21]. Carbides and nonmetallic inclusions are summarized as impurities. In fact, when non-metallic inclusions contain bubble voids, the metal internal defects caused by impurities are the cause of the fatigue failure. Such defects cause stress concentration and produce dangerous points in the cyclic stress process, leading to crack initiation and acceleration of fatigue failure [22]. It is indicated that there is a competitive process in the transition from the surface of the sub-surface of the fatigue crack, and the plastic deformation of the local cycle with the cyclic stress as the medium plays a decisive role along with its occurrence conditions. The yield strength is considered the limit, and when the cyclic stress level applied to the material is higher than the yield point, the material may be damaged by the cyclic plastic deformation. As the source of fatigue failure, the crack source region usually has a defect of stress concentration point, leading to the crack initiation. The development speed of this stage is at a relatively low level. In most cases, the surface morphology is relatively smooth and the crack initiation point is on the center line of the semicircle or ellipse region, but the area is relatively small. Such as in the case of internal cracks or large surface scratches, it is generally difficult to observe obvious fatigue crack initiations in the fracture of this type of sample after the test; only the surface morphology of the expansion region and the final rupture region existed [23]. During the fatigue process of metallic material, the fatigue fracture morphologies of various fracture modes are complex and irregular, which is conducive to the rapid crack propagation. The difference between the samples will also lead to a large difference in the fracture behaviors [24].

**Figure 11:**
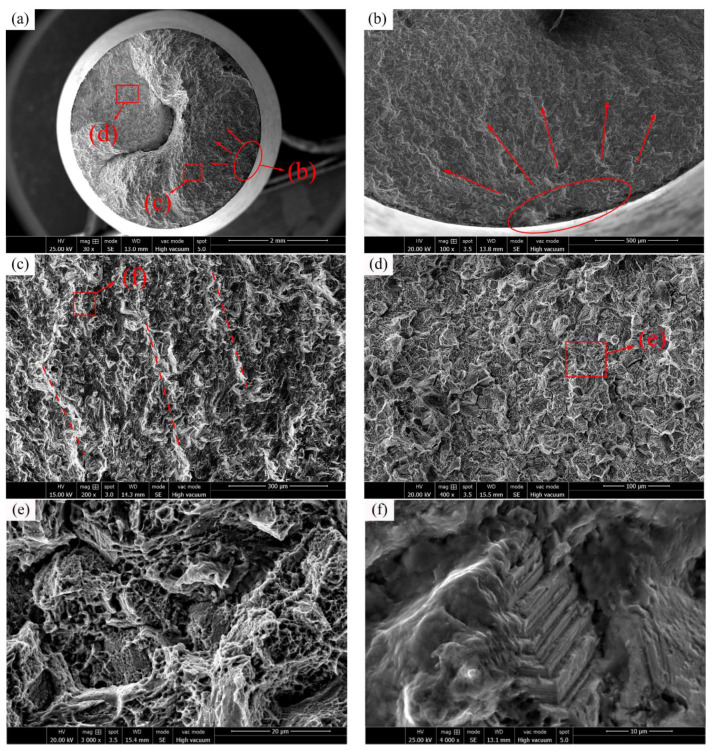
C17200 beryllium copper alloy fractured at 25 °C (*S_max_* = 490 MPa, *N_f_* = 0.46 × 10^7^ cycles). (**a**) General view of the fatigue-fractured surface; (**b**) crack initiation region; (**c**) crack propagation region; (**d**) final rupture region; (**e**) dimple fracture; and (**f**) fatigue speckle.

The typical fatigue fracture surface (*S_max_* = 490 MPa, *N_f_* = 0.46 × 10^7^ cycles) of C17200 alloy tested at 25 °C is shown in Figure 11a. It can be seen that the fracture includes three areas: crack initiation region, crack propagation region, and final rupture region. In an enlarged view of the crack initiation region, as shown in Figure 11b, it can be seen that it belongs to the surface fracture mode. It is a single source initiation, and the crack radially diffuses along the arrow direction in Figure 11b with the surface fatigue source at the center. The fatigue crack propagation region is shown in Figure 11c. The steady-state propagation region is smooth and flat with morphologies similar to that of floc, and is also known as “beach like” strips [25], and the strips are indicated by the dash lines in the Figure 11. This could be attributed the fact that the direction of the crack at the fatigue crack propagation is fronted due to different dislocation or blockage resistance under the action of periodic stress. It is formed by the intersection of fracture surfaces in different directions to form “steps” [21,26]. In the upper left corner of Figure 11a, a big crater can be observed in the final rupture region, which is usually caused by the internal crack extending radially towards deep direction. The internal fatigue crack expands and intersects under the action of periodic stress to generate cracks. In the process of crack propagation, other cracks will constantly connect the voids and produce pits of various shapes.

Moreover, Figure 11d shows the morphologies of the final rupture region. During the process of fatigue crack propagation, the propagation speed gradually expands a steady state. After reaching the critical region, the final rupture region is formed by the rapid expansion of instability, which is represented by a large number of dimples. As shown in Figure 11e, many fracture dimples can be observed in both the crack propagation region and the final rupture region, and there are more fracture dimples in the final rupture region than those in the crack propagation region [27]. Many streaks that are parallel to the extended crack can be found in the border region between the crack propagation region and the fracture region, as showed in Figure 11f. In the field of fatigue research area, such streaks are called fatigue streaks, which are formed when the fatigue crack continues to expand and can be regarded as traces of the continuous expansion of the stress cycles [28].

C17200 alloy has high tensile strength, and the main fracture mode is typical fatigue fracture, but under some special conditions will exist brittle fracture, and test temperature and applied stress levels have significant influence on the fracture mode of C17200 alloy. At 25 °C, a few C17200 alloy specimens will have brittle fracture or local brittle fracture. When brittle fracture occurs, the material is often fragmented without macroscopic plastic deformation. As the crack continues to propagate, the final stress is concentrated on a small un-cracked section until the maximum stress the section can withstand is lower than the applied stress, and the sample breaks in a short time to form a final rupture region. The morphology of the final rupture region is related to the properties of the material and external mechanical conditions, and the common feature is a rough surface.

**Figure 12:**
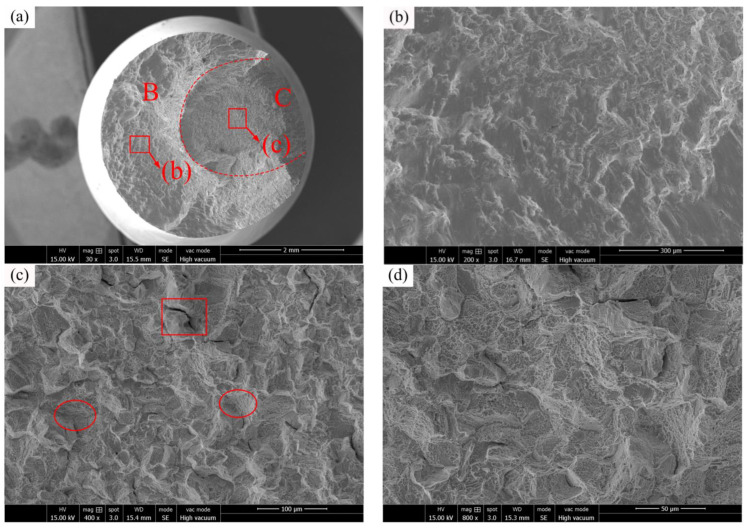
C17200 beryllium copper alloy fractured at 150 °C (*S_max_* = 450 MPa, *N_f_* = 0.43 × 10^7^ cycles). (**a**) General view of fatigue-fractured surface; (**b**) crack propagation region; (**c**) final rupture region; and (**d**) enlarged view of final rupture region.

The fatigue fracture of C17200 alloy at 150 °C (*S_max_* = 450 MPa, *N_f_* = 0.43 × 10^7^ cycles) is represented in Figure 12a. The general view of the fractured surface at 150 °C shows that there is no obvious fatigue crack initiation region; only a crack propagation region (labeled with B) and final rupture region (labeled with C) can be observed. The crack propagation region is shown in Figure 12b, where obvious “beach like” bands generated can be observed. In the process of crack propagation, if there is no well-oriented slip plane, the crack will propagate along the slip plane or the grain boundary [29]. Figure 12c shows the final rupture region, and it can be seen that there are a large number of tearing edges and dimples accompanied by brittle cracking cracks. A typical brittle cracking cracks are indicated by a red rectangle and the typical dimples are indicated by red ellipses in the Figure 12. The fracture mode belongs to a cleavage fracture mechanism [30]. Figure 12d is an enlarged figure of the specimen final rupture region, it can be observed clearly that various features were generated after the brittle fractured surface. According to Figure 11a and Figure 12a, the overall appearance of the fatigue fracture of C17200 beryllium copper alloy at 25 °C and 150 °C is uneven, and the boundary between the crack propagation region and the final rupture region is not obvious and the roughness is high, which is caused by the coarse grain of the C17200 alloy matrix and the fracture process is close to brittle fracture tearing.

Metal thermal fatigue will cause microscopic or macroscopic thermal stress in metal structure at a higher temperature, thus causing damage and destruction [31]. Macro thermal stress is the internal stress of metal materials under the action of thermal load in the non-uniform thermal fields. Microscopic thermal stress is due to the existence of inclusions within metal materials, and the expansion coefficient and elastic modulus of inclusions and matrix are usually different. After being heated, tensile force is generated between matrix and inclusion, which intensifies the separation between them.

**Figure 13:**
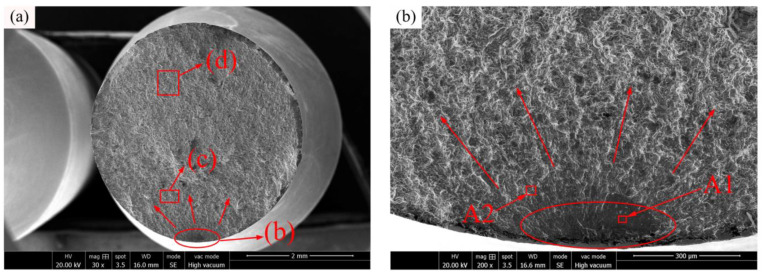

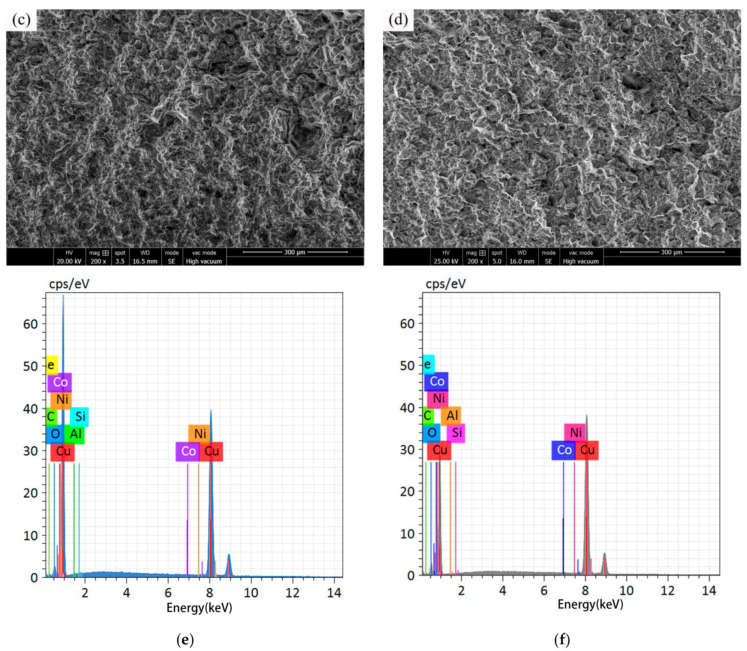
C17200 beryllium copper alloy fractured at 350 °C (*S_max_* = 420 MPa, *N_f_* = 0.31 × 10^7^ cycles). (**a**) General view of fatigue-fractured surface; (**b**) crack initiation region; (**c**) crack propagation region; (**d**) final rupture region; (**e**) EDS spectrum of point A1; and (**f**) EDS spectrum of point A2.

The typical fractured surface of the sample under RBF fracture at 350 °C (*S_max_* = 420 MPa, *N_f_* = 0.31 × 10^7^ cycles) is shown in Figure 13a. Compared to the fractured surfaces at 25 °C and 150 °C, the fractured surface is relatively flat and the surface morphology is clear. Normally, the smaller grain sizes may produce a more uniform deformation, leading to a delay of crack nucleation by reducing stress concentration. In addition, the interaction between propagated cracks and grain boundary structures would result in a delay of crack growth rate. Different fractured surface morphologies can be observed in the actual process due to the combined action of ambient temperature and different stress levels on the different materials [32]. Based on the comparison of Figure 12c and Figure 13d, it can be seen that because the grain of C17200 alloy is refined under the action of high temperature, leading to the reduction in the dimple diameter in the final rupture region, and the number of cleavage fracture plane is reduced, so that the fracture plane no longer behaves a tearing feature. In Figure 13b, it can be observed that the relatively dark and dense crack initiation area is a typical stress concentration caused by metal internal inclusions. Under the action of cyclic tension–compression stress during the RBF test, a crack initiation is then generated, and the thermal load of the metal aggravates the process of crack generation. Figure 13e,f illustrate the EDS spectra of the points A1 and A2, respectively. The results of EDS element content are listed in Table 3. It can be seen that the content of oxygen is increased at both the two points, indicating that the fracture initiation tends to be suffered oxidation, leading to an aggravation of the crack growth in some way. As showed in Figure 13c, compared with the fuzzy boundaries of the fatigue bands at a lower temperature, plastic deformation occurs to a certain extent.

Compared to the fatigue performance at 25 °C, the fatigue resistance of C17200 alloy is decreased significantly at 450 °C, which could be attributed to the fact that material is severely oxidized at such a high temperature, the mechanical properties of the sample are decreased sharply, and the thermal stress promotes the initiation of cracks and accelerates the propagation of cracks. The combination of mechanical load and environmental factors causes the cracks to propagate continuously. Larger cracks produce a higher stress concentration, which causes an increase in the crack propagation rate [33].

**Figure 14:**
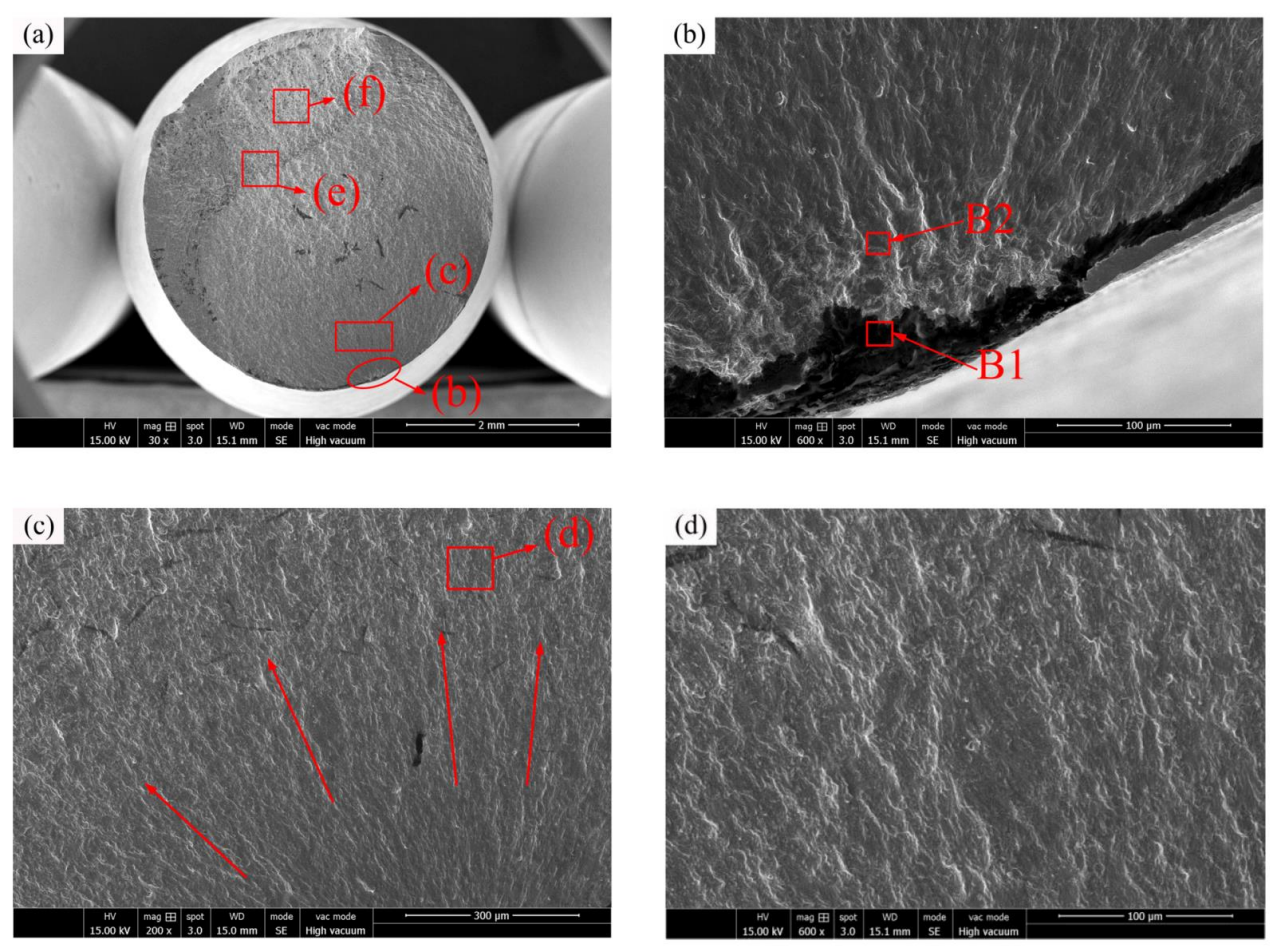

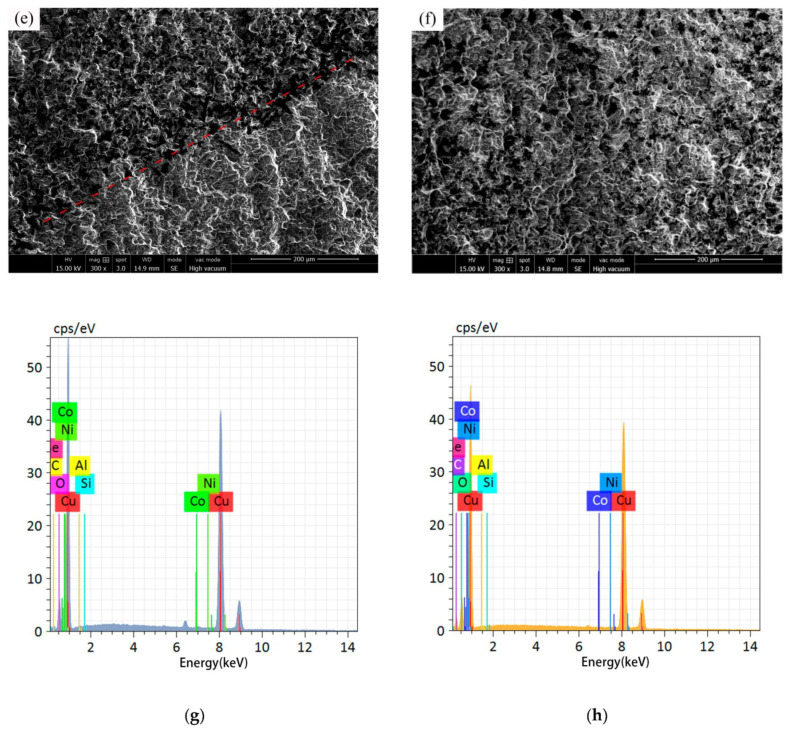
C17200 beryllium copper alloy fractured at 450 °C (*S_max_* = 270 MPa, *N_f_* = 0.64 × 10^7^ cycles). (**a**) General view of fatigue-fractured surface; (**b**) crack initiation region; (**c**) crack propagation region; (**d**) enlarged view of crack propagation region; (**e**) region of junction; (**f**) final rupture region; (**g**) EDS spectrum of point B1; and (**h**) EDS spectrum of point B2.

Figure 14a shows the typical fatigue-fractured surface of C17200 alloy specimen at 450 °C (*S_max_* = 270 MPa, *N_f_* = 0.64 × 10^7^ cycles). Compared to Figure 13a, the fractured surface is smoother, and the oxidation of the fracture is more pronounced. This is because the actual tensile yield stress of C17200 material is weakened, and the small internal cracks are constantly generated and merged under the action of the cyclic stress. The crack initiation region is shown in Figure 14b. The fatigue initiation position firstly occurred at the inclusion of the specimen. Due to plastic deformation, extensive, nearly equiaxial, dimples and cavities are generated externally from the fatigue origin, which is a typical surface fracture mode [34]. According to Figure 14g,h, oxidation at the crack source is much more severe. The comparison of oxygen content in Table 3 and Table 4 shows that the oxidation at the crack source is further intensified at 450 °C, leading to the acceleration of the crack propagation to a certain extent. The fatigue life of C17200 alloy is further reduced under the action of thermal stress and crack source oxidation. As shown in Figure 14c, the crack propagation region radiates in a fan-shaped way, with relatively uniform expansion patterns and slow development rate. The thinning density of the wave patterns in the extension region is directly related to the variation frequency of the external environment and the internal crack growth rate. Typical wavy patterns are generally associated with external load changes in high-cycle fatigue fractures at low stress levels, as shown in Figure 14d. According to Figure 14e, there is a clear dividing line (as indicated by the red dash line) between the crack propagation region and the final rupture region. The propagation region is smooth and only a few dimples appear, while the final rupture region has many holes and dimples tearing edges, which are caused by the aggregation of near-spherical particles and the particles falling off under the action of external loads.

At 450 °C, the ultimate tensile strength of C17200 alloy is decreased with a higher variation amplitude than that at 25 °C, and it can be found from the S-N curve that the fatigue strength is also obviously decreased. This is because the mechanical properties of C17200 alloy are decreased at high temperatures, and the thermal fatigue in the metal promotes the initiation of cracks. High temperature weakened the inter-grain force, accelerated the generation of fatigue speckles, significantly increased the crack propagation rate, and weakened the fatigue strength of the material [35]. Based on the above analysis, it can be seen that the mechanical properties and fatigue properties of C17200 alloy are decreased slightly when the test temperature below 350 °C, and the mechanical properties and fatigue properties are decreased severely when the test temperature reaches 450 °C. Therefore, in practical engineering applications, the C17200 alloy is more suitable for application in the environment temperature below 350 °C.

## 4. Conclusions

In this paper, tensile strength and rotating bending fatigue (RBF) tests were carried out on C17200 beryllium copper alloy at different temperatures, and fatigue fracture mechanism analysis was conducted on the specimens to explore the influence of temperature on its fatigue resistance. The following conclusions could be obtained:

(1) C17200 beryllium copper alloy had a phase transition between 400 °C and 450 °C, and the high-temperature environment reduced the force between the grain boundaries, resulting in a significant decline in the material mechanical properties at 450 °C.
(2) The tensile fracture mechanism of C17200 alloy at different temperatures was a mixture of ductile and brittle fracture mode. With the increase in temperature, the grain boundary slip surface was increased, the number of dimples was decreased, and the fracture plane tended to be flat.
(3) The RBF fatigue strength of C17200 alloy of 1 × 10^7^ cycles at 25 °C, 150 °C, 350 °C, and 450 °C was 441.8 MPa, 409.4 MPa, 400.3 MPa, and 272.1 MPa, respectively. At these four temperature gradients, the fatigue strength of C17200 alloy generally decreased with the increase in temperature. Compared to fatigue strength (1 × 10^7^ cycles) at 25 °C, the reduction amplitude of fatigue strength at 150 °C and 350 °C is within 10%; however, it is decreased by 38.4% at 450 °C.
(4) The fatigue failure type of C17200 alloy was mainly surface defect initiation at different temperatures. With the increase in temperature, the thermal stress in the specimen promoted the initiation of cracks and accelerated the propagation of cracks.

## Figures and Tables

**Table 1:** Chemical compositions of C17200 beryllium copper alloy (wt. %).

Materials	Be	Co	Al	Si	Ni	Cu
C17200 alloy	1.8–2.1	0.35–0.65	0.08–0.10	0.10–0.15	0.20–0.25	Balance

**Table 2:** Main mechanical physical properties of C17200 beryllium copper alloy [10].

Tensile Strength (MPa)	Density (g/cm^3^)	Modulus of Elasticity (GPa)	Hardness (HRC)	Thermal Conductivity (W/m·k 20 °C)	Electrical Conductivity (IACS%)
1105	8.3	128	38–44	105	18

**Table 3:** Element content of point A1 and A2 (wt. %).

Element Content	Cu	C	O	Si	Al
Point A1	85.87	6.93	5.57	0.73	0.90
Point A2	87.33	3.76	6.96	0.65	1.30

**Table 4:** Element content of point B1 and B2 (wt. %).

Element Content	Cu	C	O	Si	Al	Ni	Co
Point B1	79.36	7.01	12.12	0.33	1.11	0.07	0
Point B2	70.48	13.75	13.57	0.86	1.27	0	0.06

## Data Availability

Not applicable.
